# Assessing bias in demographic estimates from joint live and dead encounter models

**DOI:** 10.7717/peerj.9382

**Published:** 2020-06-23

**Authors:** Mitch D. Weegman, Scott Wilson, Ray T. Alisauskas, Dana K. Kellett

**Affiliations:** 1 School of Natural Resources, University of Missouri, Columbia, MO, USA; 2 Science and Technology Branch, Pacific Wildlife Research Centre, Environment and Climate Change Canada, Delta, BC, Canada; 3 Department of Forest and Conservation Sciences, University of British Columbia, Vancouver, BC, Canada; 4 Science and Technology Branch, Prairie and Northern Wildlife Research Centre, Environment and Climate Change Canada, Saskatoon, SK, Canada; 5 Department of Biology, University of Saskatchewan, Saskatoon, SK, Canada

**Keywords:** Avian ecology, Bayesian multistate joint encounter model, Capture history, Dead recovery model, M-array, Simulated data, Snow goose

## Abstract

Joint encounter (JE) models estimate demographic rates using live recapture and dead recovery data. The extent to which limited recapture or recovery data can hinder estimation in JE models is not completely understood. Yet limited data are common in ecological research. We designed a series of simulations using Bayesian multistate JE models that spanned a large range of potential recapture probabilities (0.01–0.90) and two reported mortality probabilities (0.10, 0.19). We calculated bias by comparing estimates against known probabilities of survival, fidelity and reported mortality. We explored whether sparse data (i.e., recapture probabilities <0.02) compromised inference about survival by comparing estimates from dead recovery (DR) and JE models using an 18-year data set from a migratory bird, the lesser snow goose (*Anser caerulescens caerulescens*). Our simulations showed that bias in probabilities of survival, fidelity and reported mortality was relatively low across a large range of recapture probabilities, except when recapture and reported mortality probabilities were both lowest. While bias in fidelity probability was similar across all recapture probabilities, the root mean square error declined substantially with increased recapture probabilities for reported mortality probabilities of 0.10 or 0.19, as expected. In our case study, annual survival probabilities for adult female snow geese were similar whether estimated with JE or DR models, but more precise from JE models than those from DR models. Thus, our simulated and empirical data suggest acceptably minimal bias in survival, fidelity or reported mortality probabilities estimated from JE models. Even a small amount of recapture information provided adequate structure for JE models, except when reported mortality probabilities were <0.10. Thus, practitioners with limited recapture data should not be discouraged from use of JE models. We recommend that ecologists incorporate other data types as frequently as analytically possible, since precision of focal parameters is improved, and additional parameters of interest can be estimated.

## Introduction

Joint encounter (JE) models provide a unified framework for ecologists and evolutionary biologists to incorporate information from live recaptures and dead recoveries from the same sample of marked organisms (Burnham, 1993; Lebreton et al., 1995). Provided that dead recovery (DR) data are obtained throughout the entire range of a species, such models permit estimation of probabilities for true survival (*S*), fidelity to the capture-recapture study area (*F*), recapture (*p*) and reported mortality (*r*). Reported mortality is defined as the probability that a marked animal is found dead and reported, and mathematically expressed as $r = \textstyle{f \over {\left( {1 - S} \right)}}$ where *f* is the Brownie et al. (1985) probability of recovery (White, Cordes & Arnold, 2013); Seber’s (1970) parameterization used λ as notation for *r*. Information entered into JE models includes whether an individual is seen alive, reported dead or not seen during a defined sampling period, and thus imperfect detection can be explicitly modelled and accounted for. Ecologists have adapted these models to the Bayesian and/or multistate frameworks (Barker, White & McDougall, 2005; Kendall, Conn & Hines, 2006; Kéry & Schaub, 2012; Lebreton, Almeras & Pradel, 1999), which permits inclusion of prior information as well as calculation of probabilities from state transition and observation matrices for more robust estimation. Thus, recent JE models are flexible for a variety of objectives and research questions.

Although JE models rely on live recapture and DR data, the extent to which limited information in one or both data types could hinder estimation of demographic rates remains unclear. For example, there are few published studies illustrating the influence of limited recapture data on fidelity estimation or precision of survival estimation (where strong information from multiple data types could improve precision) in JE models. Barker & Kavalieris (2001) demonstrated improvement in precision of survival estimates when recapture and resighting data were jointly modeled, but their work was largely theoretical and did not include recovery data. A potential problem of including recovery data is low average mortality in some species or limited opportunities for the recovery of dead individuals. Both of these scenarios can lead to infrequently-collected recovery data (i.e., low sample size) and poorly-estimated survival and recovery probabilities. In these cases, survival estimates could be bolstered by recapture data, depending on number of marks released and model complexity. Sparse or missing data are common in ecological research. Thus, there exists a need to understand the extent to which data limitations influence estimation and bias of demographic rates in the JE framework across a gradient of parameter space.

We were motivated to understand the influence of data limitations on bias and precision of estimates from JE models because we wanted to quantify the relative contribution of demographic rates to population growth rate in an Arctic-nesting migratory bird, the lesser snow goose (*Anser caerulescens caerulescens*; hereafter snow goose). Between the 1980s and late 2000s, snow goose populations increased rapidly in the central Canadian Arctic (Alisauskas et al., 2011), prompting concern about the negative impacts to Arctic fauna and flora (Batt, 1997). More recently, snow goose population growth rates have stabilized (Alisauskas et al., 2011) and the demographic mechanisms behind this development are unclear, although declining recruitment is suspected (Ross et al., 2017; Ross et al., 2018). Another hypothesis for stability is increased permanent movement of birds among numerous breeding colonies that range from Alaska to the eastern Canadian Arctic, which can be tested through estimation of a fidelity probability. While fidelity estimation is not possible in DR models, it is a feature of JE models using recapture data. Importantly, snow geese have been recaptured in the central Canadian Arctic over the period of population increase and stabilization. While probabilities of reported mortality by snow geese across North America are moderate (i.e., 0.18–0.20), annual recapture probabilities at Canadian Arctic banding sites are low (i.e., <0.02, Wilson, Alisauskas & Kellett, 2016), and similar to other waterfowl banded in North America (Doherty et al., 2002); low recapture probabilities could bias demographic rate estimation, particularly for probabilities such as fidelity that are directly informed by recaptures. Conservation planning for snow geese and many waterfowl species is often based on rich DR data, even though limited recapture data are often available as well. Thus, quantifying the “value added” of limited recapture data to estimation of otherwise inestimable probabilities such as fidelity and jointly estimable probabilities such as survival could better inform practitioner decision-making and conservation application.

We designed a series of simulations using JE models that spanned a large range of potential *p* (i.e., 0.01–0.90) and several *r* (i.e., 0.10–0.19). We calculated bias in *S*, *F* and *r* to understand the extent to which limited recapture data restricted estimation and inference capacity. We expected that (1) *F* would be less precise and more biased at low recaptures, and this would be most pronounced at lower *r*, (2) *S* and *r* would be estimable because of recoveries, and (3) *S* would be more precise and less biased with increased *p*. We then developed JE and DR models using an 18-year data set for snow geese to estimate *F*, and compare *S* from models with and without recapture information to determine if precision was improved when recapture data were included. We expected little improvement in *S* for the JE model relative to the DR model because previous analyses indicated *p* for adult female snow geese was very low (Wilson, Alisauskas & Kellett, 2016).

## Materials and Methods

### Simulations

We simulated individual capture histories for 100 individuals marked annually for 10 years using mean (and annual standard deviation) *S* = 0.90 (0.02), *F* = 0.90 (0.07) and *r* = 0.10 (0.02) and 0.19 (0.02) based on demographic rates for snow geese estimated from previous analyses (Wilson, Alisauskas & Kellett, 2016), and *p* = 0.01, 0.20, 0.50 and 0.90. Values for each probability were simulated using a normal distribution on the logit scale and then back-transformed to generate year-specific probabilities for each of the 10 years as follows: *S* = {0.93, 0.88, 0.91, 0.74, 0.90, 0.86, 0.95, 0.90, 0.82}, *F* = {0.84, 0.93, 0.65, 0.83, 0.72, 0.99, 0.90, 0.74, 0.89}, *r* (0.10) = {0.10, 0.06, 0.13, 0.09, 0.12, 0.13, 0.11, 0.08, 0.10}, *r* (0.19) = {0.22, 0.24, 0.17, 0.17, 0.20, 0.22, 0.20, 0.19, 0.14}, *p* (0.01) = {0.03, 0.01, 0.01, 0.01, 0.01, 0.01, 0.01, 0.02, 0.01}, *p* (0.20) = {0.31, 0.08, 0.13, 0.17, 0.36, 0.16, 0.21, 0.31, 0.20}, *p* (0.50) = {0.64, 0.37, 0.27, 0.57, 0.63, 0.54, 0.64, 0.33, 0.59} and *p* (0.90) = {0.91, 0.94, 0.85, 0.84, 0.93, 0.92, 0.88, 0.92, 0.93}. We formed eight possible combinations of *S*, *F*, *r* and *p* (e.g., combination 1: *S*, *F*, *r* = 0.10, *p* = 0.01), and used the same generated values for all simulations. We formed JE models in a Bayesian multistate framework using simulated data to quantify the influence of variable recapture and reported mortality information on estimation of four probabilities pertinent to local demography: survival, fidelity, recapture and reported mortality. We chose minimally informative priors with uniform distributions for *F* ($U\left( {0,1} \right)$), *S* ($U\left( {0,1} \right)$), *p* ($U\left( {0,1} \right)$) and *r* ($U\left( {0,1} \right)$) parameters. We included random time effects on all parameters, using a Normal distribution with mean 0 and variance σ^2^. We used a state-space formulation for JE models comprised of state transition and observation matrices. All simulations involved identical model structure including four possible states: “alive in study area”, “alive outside study area”, “recently dead” and “dead”. In this framework, only recently dead individuals could be recovered. We considered the true state of individual *i* at time *t* as ${z_{i,t}}$. We assumed no error in state assignment, and thus the first encounter, $f{s_i}$, equaled the observed state at first encounter (i.e., ${z_{i,{f_i}}}$= $f{s_i}$, where ${f_i}$ was first encounter of individual *i*). Our state transition matrix (Ω) was four-dimensional, composed of the current state, future state, individual *i* and time *t*. Thus, we calculated the probability that individual *i* was in state *a* at time *t* and state *b* at time *t* + 1 according to:

$${z_{i,t + 1}}|{z_{i,t}}{\sim}\;{\rm categorical}\;\left( {{{ \bf{\Omega }}_{{z_{i,t}},1 \ldots s,i,t}}} \right)$$

where *s* represented the four possible states. We used a categorical distribution for a vector of a complete row of Ω for given values of dimensions ${z_{i,t}}$, *i* and *t*. Following Kéry & Schaub (2012), Ω included survival, fidelity and reported mortality, and modelled all possible probabilities of transitioning between states from time *t* to time *t* + 1 (Box 1).

**Box 1:** The state transition matrix associated with our simulations representing probabilities of transitioning from a true state at time *t* to a true state at time *t* + 1. Parameters included survival (*S*), fidelity (*F*) and reported mortality (*r*) probability.

True state at time *t*	True state at time *t* + 1			
	Alive, inside	Alive, outside	Recently dead	Dead
Alive, inside	SF	*S* (1 − *F*)	(1 − *S*) *r*	(1 − *S*) (1 − *r*)
Alive, outside	0	*S*	(1 − *S*) *r*	(1 − *S*) (1 − *r*)
Recently dead	0	0	0	1
Dead	0	0	0	1

Our observation matrix (${\Theta }$) linked possible and observed states and contained three possible observable states: “seen alive”, “recovered dead” and “not seen or recovered” with recapture according to:

$${y_{i,t}}|{z_{i,t}}{\sim}\;{\rm categorical}\; \left( {{{ \Theta }_{{z_{i,t}},1 \ldots o,i,t}}} \right)$$

where *y* comprised the simulated JE data and *o* represented 3 observable states (Box 2). We used a categorical distribution for a vector of a complete row of ${ \Theta }$ for given values of dimensions ${z_{i,t}}$, *i* and *t*. We included *r* in the Ω instead of the ${ \Theta }$ to overcome an update problem, as described in Kéry & Schaub (2012).

**Box 2:** The observation matrix associated with our simulations representing the linkage between true and observed states at time *t*, and including parameter recapture probability (*p*).

True state at time *t*	Observed state at time *t* + 1		
	Seen alive	Recovered dead	Not seen or recovered
Alive, inside	*p*	0	1 − *p*
Alive, outside	0	0	1
Recently dead	0	1	0
Dead	0	0	1

We ran JE models using program JAGS (Plummer, 2003) through package jagsUI (Kellner, 2014) in Program R (R Development Core Team, 2016). For simulations with *r* = 0.19, we relied on 3 chains of length 40,000, with burn-in 20,000 and thin of 10, while for those with *r* = 0.10, the number of iterations and burn-in to achieve convergence depended on the recapture probability (i.e., when *p* = 0.01: 500,000 iterations with burn-in 100,000; when *p* = 0.20: 600,000 iterations with burn-in 550,000; when *p* = 0.50: 400,000 iterations with burn-in 350,000; when *p* = 0.90: 150,000 iterations with burn-in 100,000). We assessed convergence with *R*-hat (Brooks & Gelman, 1998) and visual inspection of chains, and stored posteriors when *R*-hat < 1.05 and chains were well mixed to draw inference from well-estimated parameters. We evaluated estimates by calculating bias as the difference between the mean estimated random effect values and inputted value, as well as root mean square error (RMSE, calculated as ${\rm RMSE} = \sqrt {{\rm SD}^2 + bias^2}$) from posterior distributions. We anticipated that bias and RMSE could be slightly optimistic because we evaluated the behavior of the mean using random time effects. Source code for our simulations are in the Supplemental Files.

We parameterized an additional set of simulations with reported mortality probabilities 0.02 and 0.05, and recapture probabilities 0.01, 0.20, 0.50 and 0.90, with all other model specifications identical to our other simulations. We experienced convergence difficulties (e.g., *R*-hat > 1.75) in survival, fidelity and reported mortality probabilities in all of these additional models, even with informed priors. Thus, researchers with recapture data but only sparse recovery data may consider using capture-recapture models instead of JE models.

### Snow geese

During August from 1997 to 2014, we caught 18,409 adult female snow geese during their flightless phase north of Karrak Lake in the Queen Maud Gulf (Ahiak) Migratory Bird Sanctuary in Nunavut, Canada (67° 14′ N, 100° 15′ W; Alisauskas et al., 2012; Slattery & Alisauskas, 2007; Wilson, Alisauskas & Kellett, 2016). We marked individuals with metal leg bands engraved with a unique numeric identifier permitted from the U.S. Geological Survey/Canadian Wildlife Service. We followed all animal marking protocols in capturing, handling and banding birds (Canadian Council on Animal Care permit number 19960014, Canadian Wildlife Service bird banding permit number 10569, both to RTA). Marked individuals could be recaptured during subsequent annual banding operations and/or recovered when harvested by hunters and reported to the Bird Banding Laboratory (see Table 1 for annual captures, recaptures and recoveries).

**Table 1:** The number of snow goose captures (releases), recaptures and recoveries per year, 1997–2014, near Karrak Lake, Nunavut, Canada.

Year	Captures	Recaptures	Recoveries
1997	384	0	0
1998	808	1	5
1999	383	15	22
2000	522	11	33
2001	576	21	44
2002	502	18	47
2003	1373	16	48
2004	1773	43	71
2005	1410	90	109
2006	555	46	112
2007	1323	56	93
2008	1134	57	115
2009	1252	90	115
2010	1692	105	91
2011	1255	45	151
2012	1077	79	152
2013	1081	80	188
2014	1309	119	192

To quantify snow goose “baseline” survival (i.e., in the absence of limited recapture data), we formed a Bayesian DR model with Seber parameterization that estimated time-dependent survival and reported mortality probabilities (Brownie et al., 1985; Seber, 1970). For improved computational efficiency and model run time, we summarized snow goose captures and reported mortalities in the m-array format, whereby rows were capture years and columns were reported mortality years (Burnham et al., 1987; Williams, Nichols & Conroy, 2002). We computed posterior distributions from the DR model using program JAGS (Plummer, 2003) through package jagsUI (Kellner, 2014) in Program R (R Development Core Team, 2016). We chose priors with uniform distributions informed by snow goose demography for survival ($S\;{\sim}\; U\left( {0,1} \right)$) and reported mortality ($r\;{\sim}\;U\left( {0.1,0.4} \right)$) parameters (Wilson, Alisauskas & Kellett, 2016), and 3 chains of length 50,000, burn-in of 25,000 and thin of 10. As in simulations, we assessed convergence using *R*-hat (Brooks & Gelman, 1998) and visual inspection of chains, and stored posteriors when *R*-hat < 1.05 and chains were well mixed to draw inference from well-estimated parameters.

Building on the DR model, we formed a Bayesian JE model to estimate snow goose survival, fidelity, recapture and reported mortality probabilities. This permitted comparisons of the posterior distributions of survival and reported mortality probabilities between DR and JE models. We chose priors with uniform distributions based on previously reported snow goose demography ($F\;{\sim}\;U\left( {0.5,1} \right)$, $S\;{\sim}\;U\left( {0.5,1} \right)$, $p\;{\sim}\;U\left( {0,0.3} \right)$ and $r\;{\sim}\; U\left( {0,0.5} \right)$; Wilson, Alisauskas & Kellett, 2016). As with our simulations, we included random time effects on all parameters, using a Normal distribution with mean 0 and variance σ^2^. Our model specification (including Ω and ${\Theta }$), convergence assessment and storage of posteriors were identical to those used in simulations, except that we used 3 chains of length 80,000, burn-in of 40,000 and thin of 10 to achieve convergence. We calculated the coefficient of variation (CV) for survival and reported mortality probabilities to compare the precision in annual estimates from JE and DR models. Source code for DR and JE models, and snow goose data are in the Supplemental Files.

## Results

### Simulations

**Figure 1:**
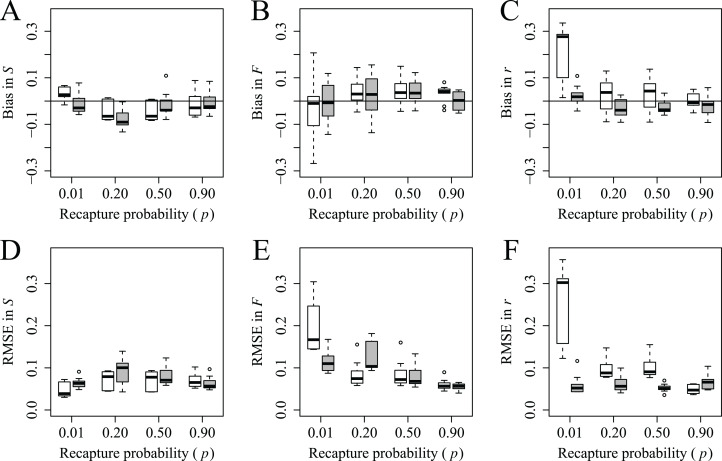
Bias and root mean square error (RMSE) in estimates of survival, *S* (A and D), fidelity, *F* (B and E) and reported mortality, *r* (C and F) probabilities across recapture and reported mortality probabilities. For each recapture probability (e.g., *p* = 0.01), results from JE models with each reported mortality probability are presented; lighter and darker boxes represent *r* = 0.10 and 0.19 respectively. Each box comprises the 25th, 50th and 75th percentiles. Whiskers represent the minimum and maximum range. Minimum range was calculated as the 25th percentile − 1.5 × inner quartile range (IQR; that is, from the 25th to 75th percentile). Maximum range was calculated as the 75th percentile + 1.5 × IQR. The points outside of the whiskers were considered outliers. Horizontal lines represent 0 bias.

Generally, bias and RMSE in survival, fidelity and reported mortality probabilities estimated from simulated data and JE models were low across our range in probabilities of recapture (i.e., 0.01–0.90) and reported mortality (i.e., 0.10–0.19). There was substantial overlap in estimates of bias and RMSE for all survival probabilities estimated from models using different recapture and reported mortality probabilities (Figs. 1A and 1D). While bias in fidelity probability was consistent and low (<0.05) across all recapture probabilities, RMSE of fidelity at the lowest recapture probabilities was much larger than at the highest probabilities of recapture, and this effect was even more pronounced at lower probabilities of reported mortality (Figs. 1B and 1E). Mean bias and RMSE in reported mortality probability were generally low and similar across the range of recapture probabilities. However, when the probability of reported mortality was low, bias and RMSE were much larger at lower probabilities of recapture and declined substantially with increased recapture probability; when *p* = 0.90, bias and RMSE were similar regardless of reported mortality probability (Figs. 1C and 1F).

### Snow geese

**Figure 2:**
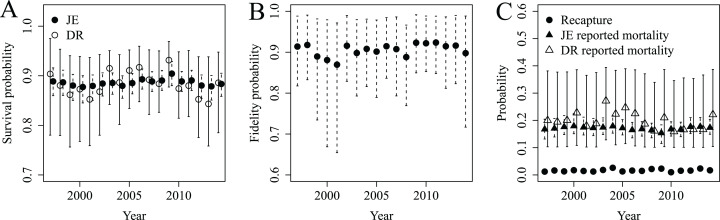
Survival (A), fidelity (B), recapture and reported mortality (C) probabilities estimated for lesser snow geese from 1997 to 2014 using JE (*S*, *F*, *r* and *p*) and DR (*S* and *r*) models. The JE and DR models were used to quantify whether precision of DR estimates could be improved by JE estimates despite low recapture probabilities (e.g., *p* = 0.02).

Annual survival probabilities for adult female snow geese were similar whether estimated with JE or DR models (mean from JE model 0.89, 95% CRI 0.86–0.91; mean from DR model 0.88, 95% CRI 0.78–0.96; Fig. 2A). However, the CV of survival estimates from the JE model were <0.02, while they were <0.07 from the DR model. Thus, survival estimates from the JE model were more precise than those from the DR model. Fidelity probability was relatively stable over time (mean 0.91, 95% CRI 0.78–0.99; Fig. 2B), as were the very low probabilities of recapture (mean 0.02, 95% CRI 0.01–0.03). Reported mortality probabilities were greater than recapture probabilities but similar between models (i.e., mean from JE model 0.17, 95% CRI 0.14–0.21; mean from DR model 0.20, 95% CRI 0.10–0.38; Fig. 2C). However, the CV for the reported mortality probability from the JE model were <0.12, while they were <0.44 from the DR model. Thus, the reported mortality probabilities from the JE model were far more precise than those from the DR model.

## Discussion

Our simulations and empirical data suggest no appreciable bias in survival, fidelity or reported mortality probabilities from Bayesian JE models, unless probabilities of recapture and reported mortality are both exceptionally low. Importantly, the addition of even limited recapture information considerably increased precision of estimated survival probabilities compared to a model that used only recovery data. The minimal bias in fidelity, even at the lowest recapture probabilities, was particularly encouraging although somewhat unexpected because fidelity is informed largely by recapture data. Simulation results for RMSE more closely matched our expectations, whereby RMSE values declined with increasing recapture probabilities, and the decline was steepest when reported mortality probability was 0.10 compared to 0.19. These results suggest that when the reported mortality probability is ≥0.10, a recapture probability of ≥0.20 provides adequate structure for practitioners to use the JE model framework efficiently. If practitioners are faced with reported mortality probability of ≥0.19, then even a small amount of recapture information (e.g., *p* = 0.01) provides increased precision in the parameter estimates. We recommend that researchers with reported mortality probability <0.19 and low recapture probabilities continue to interpret results cautiously.

While we are not aware of research explicitly evaluating bias and RMSE in JE models, our results are similar to those from recent simulation studies that explored heterogeneity using waterfowl life histories and comparable model specification with DR models (White, Cordes & Arnold, 2013; Dooley et al., 2019). White, Cordes & Arnold (2013) found that substantial variation in reporting rate did not appreciably increase bias in survival probabilities. Dooley et al. (2019) reported greater bias than we found in survival and recovery probabilities when two age classes were modeled for Canada geese (*Branta canadensis*) in the North American mid-continent, and negligible bias, similar to our findings, when three age classes were modeled. Thus, while heterogeneity in survival can be ecologically important to estimate, we do not anticipate that unmodeled heterogeneity substantially and directionally biased estimates in our application of JE models as they were based only on adult females.

The convergence of model results, and bias and precision of parameter estimates, also likely depended on sample size. We chose an annual sample size of 100 newly marked individuals for all simulations, which matched common capture-recapture projects in ecological literature (Cilimburg et al., 2002; Clark et al., 2018; Sedinger et al., 2007). We anticipate that larger sample sizes would improve convergence when probability of reported mortality is low, and greater recapture probabilities are expected to render parameters estimable. We encourage practitioners to further develop our JE models to evaluate project-specific questions and study designs. Particularly relevant future work could explore: (1) age structure, for example, parameter redundancy in age-dependent recovery models (Lakhani & Newton, 1983) and whether the addition of recapture data helps to resolve redundancy, (2) individual heterogeneity, for example, in permanent emigration to detect senescence in survival (Péron et al., 2010), and (3) sampling design, for example, to overcome identifiability issues in one or multiple data types (Freeman, Morgan & Catchpole, 1992). We recommend that researchers exploring these extensions continue to monitor bias and precision in estimates from JE models pertinent to their studies.

Results from our empirical case study with snow goose data suggest that survival and reported mortality probabilities were more precisely estimated from JE models than from DR models. In the case of survival estimation, we interpret this as further evidence of the quality and complex structure associated with recapture data. For both survival and reported mortality probabilities, we suspect that the difference in model structure between JE and DR models was responsible for differences in estimation precision; the multistate approach in our JE model used multiple data types to link true and observed states (Burnham, 1993; Lebreton et al., 1995), which were not features of our DR model. The JE model also included random time effects, with shrinkage of estimates toward the grand mean, which may further improve precision if the shrinkage removes Markov chain Monte Carlo error instead of temporally-varying process variation. Thus, in addition to minimal bias in fidelity at low recapture probabilities, practitioners can expect improved precision of survival and reported mortality probabilities for greater inference when using our multistate approach with JE models.

Our estimates of snow goose survival and fidelity are consistent with other recent analyses conducted using similar JE models for this population (e.g., in Program MARK; Calvert, Alisauskas & Kellett, 2019; Wilson, Alisauskas & Kellett, 2016). Integration of productivity data with abundance estimates and data used to estimate survival and fidelity suggests that snow goose population growth rates have stabilized in the central Canadian Arctic, and may have begun to decline more recently because of declines in fecundity and fidelity. One such hypothesis for stability is increased permanent movement of birds among numerous breeding colonies that range from Alaska to the eastern Canadian Arctic. There is recent support from multistate JE models that suggests metapopulation movements among snow goose colonies could explain much of the change in local population trajectories (R. Alisauskas, 2006–2015, personal observations). Yet our fidelity probabilities showed no substantial temporal trend. An alternative hypothesis is that declines in adult survival contributed to stabilizing population growth, but we also found no temporal trends in survival. However, our estimates were from adult females only. Adult males and juveniles are known to have greater dispersal propensities than adult females (Cooke, MacInnes & Prevett, 1975; Cooch, Rockwell & Brault, 2001; Wilson, Alisauskas & Kellett, 2016). Adults also show more stable annual survival probabilities over time compared to juveniles (Traylor et al., 2012). Thus, inclusion of data from the three other age-sex classes would permit a more robust evaluation of the extent to which either (1) dispersal or permanent emigration (i.e., the complement to fidelity) from the Karrak Lake region, or (2) declines in survival explain the recent stabilization and possibly the start of a decline in snow goose population growth rate there. If fidelity and survival estimates are representative of the other age-sex classes, then any temporal variation in permanent movements must be balanced by immigration or productivity; the former has never been estimated for this population, while the latter has been monitored and is far more variable than fidelity or survival over the study period (Ross et al., 2017, 2018). To properly evaluate drivers of snow goose demography in the central Canadian Arctic with minimal bias from low recapture probabilities, we recommend development of an integrated population model that links population dynamics with hypothesized drivers, such as cross-seasonal effects of climate and landscape change (Sedinger & Alisauskas, 2014). Such a model could provide a comprehensive demographic assessment for continued conservation planning of this population (Zipkin & Saunders, 2018). This is necessary because snow geese are still considered abundant and harvested virtually without restrictions to open seasons, daily take or hunt methods in Canada and the U.S. Recent evidence suggests that current harvest pressure has had little to no impact on the population (Alisauskas et al., 2011; Calvert, Alisauskas & White, 2017). Therefore, the role of demographic parameters other than adult survival (such as recruitment, philopatry and adult fidelity, and even metapopulation source-sink dynamics) to local population change merit greater attention.

## Conclusions

We designed a series of simulations using Bayesian multistate JE models that spanned a large range of potential recapture probabilities (0.01–0.90) and two reported mortality probabilities (0.10, 0.19). We calculated bias by comparing estimates against known probabilities of survival, fidelity and reported mortality. We also explored whether sparse data (i.e., recapture probabilities <0.02) compromised inference about survival by comparing estimates from DR and JE models using an 18-year data set from a migratory bird. Our simulated and empirical data suggest acceptably minimal bias in survival, fidelity or reported mortality probabilities estimated from JE models. Even a small amount of recapture information provided adequate structure for JE models, except when reported mortality probabilities were <0.10. We recommend that practitioners should collect both recapture and recovery data where possible to improve inference in demographic research. We emphasize the importance of recapture data for practitioners who might recapture, but not record such events in the pursuit of marking “new” individuals. We also recommend continued evaluation of bias, and the sensitivity of parameter estimates to sparse data arising from low encounters of either live or dead individually-marked animals.

## Supplemental Information

10.7717/peerj.9382/supp-1 Supplemental Information 1 Code for simulated joint encounter and snow goose dead recovery, joint encounter models.

10.7717/peerj.9382/supp-2 Supplemental Information 2 Dead recovery model snow goose data.

10.7717/peerj.9382/supp-3 Supplemental Information 3 Joint encounter model snow goose data.

## Additional Information and Declarations

### Field Study Permissions

The following information was supplied relating to field study approvals (i.e., approving body and any reference numbers): Capturing, handling and banding birds was approved by the Canadian Council on Animal Care and Canadian Wildlife Service.

### Data Availability

The following information was supplied regarding data availability: The joint encounter model code using simulated and snow goose data and dead recovery model code using snow goose data are available as Supplemental Files.

## References

[ref-1] Alisauskas RT, Drake KL, Caswell JH, Kellett DK (2012). Movement and persistence by Ross’s geese (*Chen rossii*) in Canada’s Arctic. Journal of Ornithology.

[ref-2] Alisauskas RT, Rockwell RF, Dufour KW, Cooch EG, Zimmerman G, Drake KL, Leafloor JO, Moser TJ, Reed ET (2011). Harvest, survival, and abundance of midcontinent lesser snow geese relative to population reduction efforts. Wildlife Monographs.

[ref-3] Barker RJ, Kavalieris L (2001). Efficiency gain from auxiliary data requiring additional nuisance parameters. Biometrics.

[ref-4] Barker RJ, White GC, McDougall M (2005). Movement of paradise shelduck between molt sites: a joint multistate-dead recovery mark-recapture model. Journal of Wildlife Management.

[ref-5] Batt BDJ (1997). Arctic ecosystems in peril: report of the Arctic Goose Habitat Working Group.

[ref-6] Brooks SP, Gelman A (1998). General methods for monitoring convergence of iterative simulations. Journal of Computational and Graphical Statistics.

[ref-7] Brownie C, Anderson DR, Burnham KP, Robson DS (1985). Statistical inference from band recovery data: a handbook.

[ref-8] Burnham KP, Lebreton JD (1993). A theory for combined analysis of ring recovery and recapture data. Marked Individuals in the Study of Bird Populations.

[ref-9] Burnham KP, Anderson DR, White GC, Brownie C, Pollock KH (1987). Design and analysis methods for fish survival experiments based on release-recapture. American Fisheries Society Monograph.

[ref-10] Calvert AM, Alisauskas RT, Kellett DK (2019). Fitness heterogeneity in adult snow and Ross’s geese: survival is higher in females with brood patches. Auk: Ornithological Advances.

[ref-11] Calvert AM, Alisauskas RT, White GC (2017). Annual survival and seasonal hunting mortality of midcontinent snow geese. Journal of Wildlife Management.

[ref-12] Cilimburg AB, Lindberg MS, Tewksbury JJ, Hejl SJ (2002). Effects of dispersal on survival probability of adult yellow warblers (*Dendroica petechia*). Auk.

[ref-13] Clark RG, Winkler DW, Dawson RD, Shutler D, Hussell DJT, Lombardo MP, Thorpe PA, Dunn PO, Whittingham LA (2018). Geographic variation and environmental correlates of apparent survival rates in adult tree swallows *Tachycineta bicolor*. Journal of Avian Biology.

[ref-14] Cooch E, Rockwell RF, Brault S (2001). Retrospective analysis of demographic responses to environmental change: a lesser snow goose example. Ecological Monographs.

[ref-15] Cooke F, MacInnes CD, Prevett JP (1975). Gene flow between breeding populations of lesser snow geese. Auk.

[ref-16] Doherty PF, Nichols JD, Tautin J, Voelzer JF, Smith GW, Benning DS, Bentley VR, Bidwell JK, Bollinger KS, Brazda AR, Buelna EK, Goldsberry JR, King RJ, Roetker FH, Solberg JW, Thorpe PP, Wortham JS (2002). Sources of variation in breeding-ground fidelity of mallards (*Anas platyrhynchos*). Behavioral Ecology.

[ref-17] Dooley JL, Szymanski ML, Murano RJ, Vrtiska MP, Bidrowski TF, Richardson JL, White GC (2019). Age class dynamics of Canada geese in the Central Flyway. Journal of Wildlife Management.

[ref-18] Freeman SN, Morgan BJT, Catchpole EA (1992). On the augmentation of ring recovery data with field information. Journal of Animal Ecology.

[ref-19] Kellner KF (2014). https://cran.r-project.org/web/packages/jagsUI/jagsUI.pdf.

[ref-20] Kendall WL, Conn PB, Hines JE (2006). Combining multistate capture-recapture data with tag recoveries to estimate demographic parameters. Ecology.

[ref-21] Kéry M, Schaub M (2012). Bayesian population analysis using WinBUGS: a hierarchical perspective.

[ref-22] Lakhani KH, Newton I (1983). Estimating age-specific bird survival rates from ring recoveries—can it be done?. Journal of Animal Ecology.

[ref-23] Lebreton J-D, Almeras R, Pradel R (1999). Competing events, mixtures of information and multistratum recapture models. Bird Study.

[ref-24] Lebreton J-D, Morgan BJT, Pradel R, Freeman SN (1995). A simultaneous survival rate analysis of dead recovery and live recapture data. Biometrics.

[ref-26] Péron G, Crochet P-A, Choquet R, Pradel R, Lebreton J-D, Gimenez O (2010). Capture-recapture models with heterogeneity to study survival senescence in the wild. Oikos.

[ref-25] Plummer M (2003). JAGS: a program for analysis of Bayesian graphical models using Gibbs sampling.

[ref-27] R Development Core Team (2016). R: a language and environment for statistical computing.

[ref-28] Ross MV, Alisauskas RT, Douglas DC, Kellett DK (2017). Decadal declines in avian herbivore reproduction: density-dependent nutrition and phenological mismatch in the Arctic. Ecology.

[ref-29] Ross MV, Alisauskas RT, Douglas DC, Kellett DK, Drake KL (2018). Density-dependent and phenological mismatch effects on growth and survival in lesser snow and Ross’s goslings. Journal of Avian Biology.

[ref-30] Seber GAF (1970). Estimating time-specific survival and reporting rates for adult birds from band returns. Biometrika.

[ref-31] Sedinger JS, Alisauskas RT (2014). Cross-seasonal effects and the dynamics of waterfowl populations. Wildfowl.

[ref-32] Sedinger JS, Nicolai CA, Lensink CJ, Wentworth C, Conant B (2007). Black brant harvest, density dependence, and survival: a record of population dynamics. Journal of Wildlife Management.

[ref-33] Slattery SM, Alisauskas RT (2007). Distribution and habitat use of Ross’s and lesser snow geese during late brood rearing. Journal of Wildlife Management.

[ref-34] Traylor JJ, Alisauskas RT, Slattery SM, Drake KL (2012). Comparative survival and recovery of Ross’s and Lesser Snow Geese from Canada’s Central Arctic. Journal of Wildlife Management.

[ref-35] White GC, Cordes LS, Arnold TW (2013). Band reporting rates of waterfowl: does individual heterogeneity bias estimated survival rates?. Ecology and Evolution.

[ref-36] Williams BK, Nichols JD, Conroy MJ (2002). Analysis and management of animal populations.

[ref-37] Wilson S, Alisauskas RT, Kellett DK (2016). Factors influencing emigration of Ross’s and snow geese from an Arctic breeding area. Journal of Wildlife Management.

[ref-38] Zipkin EF, Saunders SP (2018). Synthesizing multiple data types for biological conservation using integrated population models. Biological Conservation.

